# Long COVID and Cardiovascular Diseases Among U.S. Adults: Results From the U.S. Medical Expenditure Panel Survey

**DOI:** 10.1177/11795468261455387

**Published:** 2026-05-25

**Authors:** Geoffrey Y. Zhang, John C. Lin, Dang Nguyen, Sameed Ahmed M. Khatana, Thomas P. Giordano

**Affiliations:** 1 3989 Baylor College of Medicine, School of Medicine, Houston, TX, USA; 2 Perelman School of Medicine, 6572 University of Pennsylvania, Philadelphia, PA, USA; 3 Department of Epidemiology, RinggoldId: 1857 Harvard T.H. Chan School of Public Health, Harvard University, Boston, MA, USA; 4 Division of Cardiovascular Medicine, Department of Medicine, Perelman School of Medicine, 6572 University of Pennsylvania, Philadelphia, PA, USA; 5 Section of Infectious Diseases, Department of Medicine, 3989 Baylor College of Medicine, Houston, TX, USA

**Keywords:** long COVID, cardiovascular disease, epidemiology

## Abstract

**Objective:**

Long COVID is associated with persistent symptoms including cardiovascular complications; however, the epidemiology and directionality of this association remain unclear.

**Methods:**

Utilizing a retrospective cohort study design with cross-sectional analyses, 8,332 respondents aged 18 and older from the 2022 Medical Expenditure Panel Survey (MEPS) who had a prior COVID-19 infection, were analyzed to determine the temporal association between long COVID and cardiovascular disease (CVD), modeling each as both outcome and exposure in separate analyses.

**Results:**

Long COVID was associated with any CVD diagnosis (OR 1.37; 95% CI: 1.05-1.80), specifically angina (OR 1.81; 95% CI: 1.18-2.77) and myocardial infarction (OR 1.50; 95% CI: 1.01-2.23). Temporally, long COVID was associated with higher odds of CVD diagnoses in the same year or following year after COVID-19 (OR 2.62; 95% CI: 1.05–6.51) and in subsequent years only (OR 8.60; 95% CI: 1.53–48.3). Respondents with pre-existing CVD did not have statistically significant greater odds of reporting new long COVID symptoms.

**Conclusion:**

Our findings demonstrate that long COVID is associated with the subsequent development of CVD, underscoring the need for further research in this patient population to improve health interventions.

## Introduction

Long COVID, defined as persistent symptoms following acute coronavirus disease 2019 (COVID-19), has been classified by the Centers for Disease Control and Prevention as a chronic condition of significant public health concern.^1^ As of 2023, an estimated 17.8 million people in the United States have had long COVID, contributing to substantial long-term healthcare utilization.^2^

Despite its prevalence, long COVID is not well-defined, making it difficult to distinguish its impact on health from that of the sequelae of acute COVID-19.^3^ Because large, diagnosis-specific data with long COVID are sparse, outcomes from general post-infection populations are often analyzed, with many conditions subsequently attributed to long COVID. This can blur the distinction between post-acute sequelae of COVID-19—such as cardiovascular disease (CVD) including myocardial infarction, cerebrovascular disease and cardiac arrest^3,4^—and the broader manifestations of long COVID.

This relationship between long COVID and CVD remains poorly understood. Specifically, it remains unclear whether long COVID increases risk of new-onset CVD or whether pre-existing CVD raises the risk of developing long COVID. To address these gaps, we analyzed data from the 2022 Medical Expenditure Panel Survey (MEPS), a nationally representative survey conducted in the United States, to examine associations between long COVID and CVD among individuals with prior SARS-CoV-2 infection.

## Methods

This study was exempt from institutional review board oversight under 45 CFR 46.104 (“Exempt Research”) as it used publicly available, deidentified data. The MEPS is a nationally representative survey of noninstitutionalized, nonmilitary individuals in the United States with an overlapping panel design. Participants are interviewed five times over two years, with data collection occurring simultaneously for two panels in any given year. Additional details on MEPS methodology are available from the Agency for Healthcare Research and Quality.^5^

This analysis was a retrospective cohort study with cross-sectional analyses from MEPS participants in 2022, which had an overall response rate of 23.4%.^5^ Adults aged ≥ 18 at the end of 2022 who reported prior COVID-19 were included in the present study; 167 were excluded for incomplete demographic, socioeconomic, or health data.

All data were self-reported in the MEPS. Ever long COVID was defined as ever reporting symptoms lasting ≥ 3 months after COVID-19. CVD was based on a self-reported diagnosis of coronary heart disease, angina, myocardial infarction, heart failure, or stroke. Year of most recent COVID-19 diagnosis was recorded for individuals reporting SARS-CoV-2 infection within the past 12 months. Pre-existing CVD was defined as a CVD diagnosis before the year of COVID-19. New-onset CVD was assessed in two ways: diagnoses occurring in the same or following year as COVID-19; and only diagnoses occurring after the year of COVID-19.

The primary outcomes were self-reported long COVID and/or CVD. Statistical analyses were conducted with R 4.4.2 using 2022 MEPS sampling weights to account for survey design and nonresponse.^5^ Adults with and without long COVID were compared using Rao-Scott χ2 tests. Logistic regression assessed associations between any CVD and long COVID, adjusting for covariates.

To characterize the temporal association between long COVID and CVD, we fit logistic regression models two ways: first, assessing the risk of developing CVD among those with and without prior long COVID; second, assessing the risk of developing long COVID among those with and without pre-existing CVD.

## Results

The analysis included 8,332 participants with a history of COVID-19. Overall, 13.7% reported ever having long COVID and 7.4% reported any prior CVD diagnosis. Compared with those without long COVID, those with long COVID were more likely female (66.0% vs. 52.1%), aged ≥ 51 (46.4% vs. 41.2%), uninsured (6.8% vs. 4.3%), and unemployed (36.1% vs. 29.8%) (Table 1).

**Table 1.** Demographic and Health Comparison of US Adults Who Reported Ever Having COVID-19 by Long COVID Status, MEPS Full Year Consolidated 2022

Characteristics	Ever Long COVID (n = 1,205), %	Never Long COVID (n = 7,127), %	P-Value
Cardiovascular Disease			p < 0.001
No	88.13	93.22	​
Yes	11.87	6.78	​
Specific CVD condition			p < 0.001
Angina	2.53	1.57	​
CHD	3.49	3.65	​
MI	4.33	2.02	​
Stroke	4.36	3.22	​
Age			p < 0.001
18-30	13.67	20.07	​
31-50	39.92	38.77	​
51-64	26.79	21.35	​
65+	19.63	19.82	​
Sex			p < 0.001
Male	34.01	47.91	​
Female	65.99	52.09	​
Race/Ethnicity			p < 0.001
NH White Only	72.26	68.02	​
Hispanic	13.72	13.43	​
NH Black Only	6.22	7.70	​
NH Asian Only	3.83	8.27	​
NH Other/Multi-race	3.96	2.58	​
Household Income			p < 0.001
<100% FPL	12.96	6.92	​
100-199% FPL	15.82	11.17	​
200-399% FPL	34.25	25.73	​
400+% FPL	36.97	56.18	​
US Region			p = 0.197
Northeast	17.23	19.27	​
Midwest	26.36	21.31	​
South	31.95	32.65	​
West	24.47	26.77	​
Insurance			p < 0.001
Any private	66.30	76.26	​
Public only	26.87	19.48	​
Uninsured	6.82	4.25	​
Education			p < 0.001
High school or less	52.48	41.67	​
Some college<	34.74	37.78	​
Graduate or more	12.79	20.56	​
Employment			p < 0.001
No	36.12	29.76	​
Yes	63.88	70.24	​
Hypertension			p < 0.001
No	55.73	70.20	​
Yes	44.27	29.81	​
Current Smoking			p < 0.001
Not at all	86.46	91.68	​
Some Days	2.77	3.05	​
Every Day	10.77	5.27	​
Body Mass Index			p < 0.001
Normal weight	52.71	59.82	​
Overweight	19.64	20.16	​
Obese	27.66	20.02	​
COVID Vaccine			p < 0.001
Yes	81.13	89.85	​
No	18.87	10.15	​
No. chronic conditions, mean (SE)	0.83 (0.06)	0.55 (0.02)	—

*Note.* **Abbreviations:** CVD, cardiovascular disease; CHD, coronary heart disease; MI, myocardial infarction; NH, non-Hispanic; FPL, federal poverty level; SE, standard error; —, data not available.

In cross-sectional non-temporal analysis with long COVID as the dependent variable, CVD was more prevalent among individuals with long COVID (11.9%) than those without (6.8%). Long COVID was associated with increased odds of any CVD in unadjusted (OR 1.96; 95% CI: 1.56-2.46) and adjusted regression (OR 1.37; 95% CI: 1.05-1.80) (Table 2). Examining specific CVD conditions, long COVID was associated with angina (OR 1.81; 95% CI: 1.18-2.77) and myocardial infarction (OR 1.50; 95% CI: 1.01-2.23), but not with coronary heart disease (OR, 1.23; 95% CI, 0.86-1.73) or stroke (OR, 1.28; 95% CI, 0.88-1.83) in multivariable regression. Long COVID was also associated with female sex, US Midwest region, age groups 31-50 and 51-64 years old, lack of COVID vaccination, obesity, hypertension, and more chronic conditions. Non-Hispanic Black participants had lower odds of long COVID compared to non-Hispanic White participants.

**Table 2.** Prevalence and Multivariate Odds Ratios for Long COVID in Association With Cardiovascular and Other Factors Among Respondents Who Reported Ever Having COVID-19, MEPS Full Year Consolidated 2022

Characteristics (N=8,332)	Adjusted prevalence of long COVID, %	Adjusted odds ratio (95% CI) of long COVID
Cardiovascular Disease		
No	12.97	1 [reference]
Yes	22.58	**1.37 (1.05-1.80)**
Age		
18-30	9.95	1 [reference]
31-50	12.76	**1.30 (1.00-1.70)**
51-64	17.87	**1.52 (1.13-2.03)**
65+	14.81	0.93 (0.67-1.28)
Sex		
Male	10.45	1 [reference]
Female	16.53	**1.70 (1.45-2.00)**
Race/Ethnicity		
NH White Only	14.60	1 [reference]
Hispanic	13.19	0.93 (0.74, 1.17)
NH Black Only	11.60	**0.72 (0.53, 0.97)**
NH Asian Only	7.33	0.65 (0.42, 1.03)
NH Other/Multi-race	15.29	0.94 (0.59, 1.48)
Household Income		
<100% FPL	17.34	1 [reference]
100-199% FPL	17.50	1.09 (0.80, 1.49)
200-399% FPL	15.67	1.09 (0.84, 1.43)
400+% FPL	10.94	0.81 (0.60, 1.09)
US Region		
Northeast	11.67	1 [reference]
Midwest	15.53	**1.29 (1.00, 1.66)**
South	13.83	1.07 (0.83, 1.37)
West	13.28	1.22 (0.96, 1.54)
Insurance		
Any private	12.59	1 [reference]
Public only	16.54	0.93 (0.75, 1.16)
Uninsured	17.53	1.33 (0.95, 1.87)
Education		
High school or less	15.64	1 [reference]
Some college	12.97	0.89 (0.74, 1.07**)**
Master’s or more	9.22	**0.68 (0.51, 0.89)**
Employment		
No	16.17	1 [reference]
Yes	12.61	0.96 (0.79, 1.17)
Hypertension		
No	11.62	1 [reference]
Yes	18.51	**1.35 (1.13, 1.61)**
Current Smoking		
Not at all	13.21	1 [reference]
Some Days	15.20	0.94 (0.64, 1.38)
Every Day	20.14	1.20 (0.87, 1.67)
Body Mass Index		
Normal weight	12.11	1 [reference]
Overweight	13.68	1.06 (0.87,1.30)
Obese	18.21	**1.29 (1.03,1.59)**
COVID-19 Vaccination		
Yes	12.97	1 [reference]
No	17.13	**1.30 (1.00, 1.70)**
No. chronic conditions		**1.37 (1.22, 1.53)**

*Note.* Statistically significant relationships (p<0.05) are bolded.

**Abbreviations:** CVD, cardiovascular disease; CHD, congestive heart disease; MI, myocardial infarction; NH, non-Hispanic; FPL, federal poverty level; SE, standard error; —, data not available.

Data derived from a multivariable model of cross-sectional data with long COVID as the dependent variable, and the factors listed in the table as independent variables.

In temporal analysis with new CVD as the dependent variable, participants diagnosed with long COVID had higher odds of receiving a new CVD diagnosis in the same or following calendar year compared to those without long COVID (OR 2.62; 95% CI: 1.05-6.51) (Table 3A). Similarly, these participants also had higher odds of receiving a subsequent CVD diagnosis in the next calendar compared to those without long COVID (OR 8.60; 95% CI: 1.53-48.3) (Table 3B). On the other hand, in models of new long COVID, participants with pre-existing CVD did not have statistically significant greater odds of reporting new long COVID symptoms (OR 0.91; 95% CI: 0.61-1.36) (Table 3C).

**Table 3.** Long COVID in Relation to Subsequent vs. Prior CVD, Among Respondents Who Reported Ever Having COVID-19, MEPS Full Year Consolidated 2022^a^ (N = 4,328)

3A. Diagnosis of subsequent CVD in the same or following year by long COVID status			
Long COVID status	Adjusted absolute risk	Adjusted odds ratio (95% CI)	P-value
No Long COVID	0.85%	1 [reference]	—
Long COVID	2.38%	**2.62 (1.05-6.51)**	0.038
**3B. Diagnosis of subsequent CVD in the Following Year by Long COVID status**			
No Long COVID	0.10%	1 [reference]	—
Long COVID	0.84%	**8.60 (1.53-48.3)**	0.015
**3C. Development of Long COVID by CVD status with vs. without prior CVD**			
**CVD Status**	**Adjusted Absolute Risk**	**Adjusted Odds Ratio (95% CI)**	**P-Value**
No Pre-existing CVD	10.49%	1 [reference]	—
With Pre-existing CVD	16.49%	0.91 (0.61-1.36)	0.63

*Note.* All data derived from a multivariable model with Tables A and B using subsequent CVD as the dependent variable, and long COVID and the independent variable. Table C used subsequent Long COVID as the dependent variable and pre-existing CVD factors as the independent variable.

^a^Estimates adjusted for age, sex, household income, region, education, smoking history, insurance, employment, weight status, number of COVID vaccinations, and other chronic conditions (arthritis, asthma, cancer, COPD, diabetes, hypertension).

## Discussion

In this cohort study of 8,332 MEPS participants, long COVID was associated with higher odds of subsequent CVD. Pre-existing CVD was not associated with higher odds of subsequent long COVID. This aligns with prior research showing elevated cardiovascular risk following acute COVID-19.^6^ While this risk may reflect more severe or prolonged acute infection in those who subsequently develop long COVID, growing evidence suggests long COVID and CVD share underlying biological mechanisms, including chronic inflammation, endothelial dysfunction, and immune dysregulation.^6-8^ These findings indicate that long COVID may not simply be a marker of severe infection but could represent a distinct post-viral syndrome with cardiovascular implications.

Long COVID continues to emerge in patients infected during recent COVID-19 waves, despite lower acute severity, and shares features with other post-viral syndromes.^9^ This suggests long COVID will remain a public health concern even as the number of acute COVID-19 cases declines. The potential for long-term cardiovascular complications underscores the need for sustained clinical vigilance and multidisciplinary care, particularly for higher-risk populations, including women, older adults, and socioeconomically disadvantaged individuals.^10^ Clinical care for individuals with long COVID should include cardiovascular risk assessment, particularly in those presenting with respiratory symptoms, chest pain, or other cardiopulmonary concerns, as recommended by guidelines.^11,12^

This study has several limitations. Long COVID and other dates of diagnoses were self-reported. The definition of long COVID may lack specificity, as symptoms overlap with those of CVD and diagnostic criteria have changed over time. Our sample size was constrained by the available MEPS cohort with no formal power analysis performed. The absence of exact diagnosis dates beyond the year of diagnosis prevented precise temporal analyses. The one-year data window limited assessment of longer-term outcomes. Analyses of CVD subtypes were limited by small numbers of participants with both long COVID and CVD. Findings may be affected by detection bias due to potential differences in healthcare utilization between those with and without long COVID, potentially influencing diagnosis or recording of cardiovascular conditions.

## Conclusion

This is the first nationally representative cohort study examining the association between long COVID and CVD. In this nationally representative sample of adults with a history of COVID-19, long COVID was associated with higher odds of cardiovascular disease, particularly angina and myocardial infarction, and with a greater likelihood of subsequent CVD diagnosis in the same or following year. By contrast, pre-existing cardiovascular disease was not clearly associated with an increased risk of reporting new long COVID symptoms. These findings suggest that long COVID may be an important marker of cardiovascular risk rather than merely a reflection of prior CVD and highlight the need for ongoing cardiovascular monitoring and supportive care in individuals with long COVID, especially those with additional sociodemographic and cardiometabolic risk factors. Future research should explore causal mechanisms and long-term outcomes. Continued attention to the cardiovascular consequences of long COVID is essential to address its enduring impact on population health.

## Data Availability

All data and documentation for the Medical Expenditure Panel Survey are available on websites published by the Agency for Healthcare Research and Quality: https://www.meps.ahrq.gov/mepsweb
